# Crystal structure of 2-amino-5-methyl­sulfanyl-1,3,4-thia­diazol-3-ium chloride monohydrate

**DOI:** 10.1107/S1600536814015864

**Published:** 2014-08-01

**Authors:** Sarra Soudani, Emmanuel Aubert, Christian Jelsch, Cherif Ben Nasr

**Affiliations:** a Laboratoire de Chimie des Matériaux, Faculté des sciences de Bizerte, 7021 Zarzouna, Tunisia; b Cristallographie, Résonance Magnétique et Modélisations (CRM2), UMR CNRS–UHP 7036, Institut Jean Barriol, Université de Lorraine, BP 70239, Boulevard des Aiguillettes, 54506 Vandoeuvre-les-Nancy, France

**Keywords:** crystal structure, 1,3,4-thia­diazole, biological activity, organic-inorganic hybrid

## Abstract

The title salt, C_3_H_6_N_3_S_2_^+^·Cl^−^·H_2_O, crystallized with two organic cations, two chloride anions and two water mol­ecules in the asymmetric unit. The methyl C atoms deviate from their respective bound ring planes by 0.081 and 0.002 Å. In the crystal, the components are connected *via* N—H⋯O, N—H⋯Cl and O—H⋯Cl hydrogen bonds, forming sheets lying parallel to (100). The sheets are linked into bilayers by O—H⋯Cl hydrogen bonds involving the chloride ions and water mol­ecules. Within the bilayers there are π–π inter­actions [inter-centroid distances = 3.4654 (4) and 3.4789 (4) Å] involving inversion-related cations.

## Related literature   

For the medicinal importance and biological activity of thia­diazol isomers, see: Demirbas *et al.* (2009 ▶). For applications of 1,3,4 thia­diazo­les in agriculture, see: Wei *et al.* (2006 ▶). For C—N bond lengths in the 2-amino-5-methyl­sulfanyl-1,3,4-thia­diazol-3-ium cation, see: Mrad *et al.* (2012 ▶).
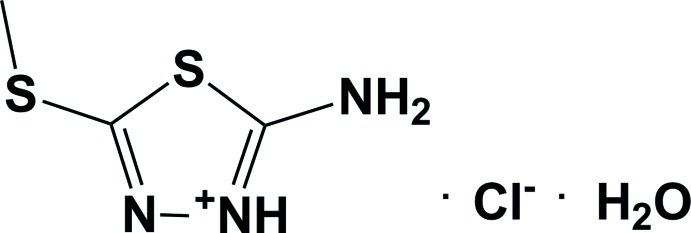


## Experimental   

### Crystal data  

C_3_H_6_N_3_S_2_^+^·Cl^−^·H_2_O
*M* *_r_* = 201.69
Monoclinic, 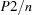

*a* = 13.3826 (2) Å
*b* = 9.4258 (1) Å
*c* = 13.5762 (2) Å
β = 99.453 (1)°
*V* = 1689.27 (4) Å^3^
*Z* = 8
Mo *K*α radiation
μ = 0.89 mm^−1^
*T* = 110 K
0.42 × 0.31 × 0.15 mm

### Data collection  

Agilent SuperNova (Dual, Cu at zero, Atlas) diffractometer
Absorption correction: analytical [*CrysAlis PRO* (Agilent, 2012 ▶), using a multi-faceted crystal model based on expressions derived by Clark & Reid (1995 ▶)] *T*_min_ = 0.769, *T*_max_ = 0.894
65950 measured reflections
8394 independent reflections
7745 reflections with *I* > 2σ(*I*)
*R*_int_ = 0.021

### Refinement  

*R*[*F* ^2^ > 2σ(*F* ^2^)] = 0.020
*wR*(*F* ^2^) = 0.054
*S* = 1.12
8394 reflections
224 parameters
H atoms treated by a mixture of independent and constrained refinement
Δρ_max_ = 0.47 e Å^−3^
Δρ_min_ = −0.28 e Å^−3^

### 

Data collection: *CrysAlis PRO* (Agilent, 2012 ▶); cell refinement: *CrysAlis PRO*; data reduction: *CrysAlis PRO*; program(s) used to solve structure: *SIR92* (Altomare *et al.*, 1994 ▶); program(s) used to refine structure: *SHELXL97* (Sheldrick, 2008 ▶); molecular graphics: *ORTEP-3 for Windows* (Farrugia, 2012 ▶); software used to prepare material for publication: *SHELXL97* and *PLATON* (Spek, 2009 ▶).

## Supplementary Material

Crystal structure: contains datablock(s) global, I. DOI: 10.1107/S1600536814015864/su2749sup1.cif

Structure factors: contains datablock(s) I. DOI: 10.1107/S1600536814015864/su2749Isup2.hkl

Supporting information file. DOI: 10.1107/S1600536814015864/su2749Isup3.tif

Supporting information file. DOI: 10.1107/S1600536814015864/su2749Isup4.cml

**Fig. 1.**
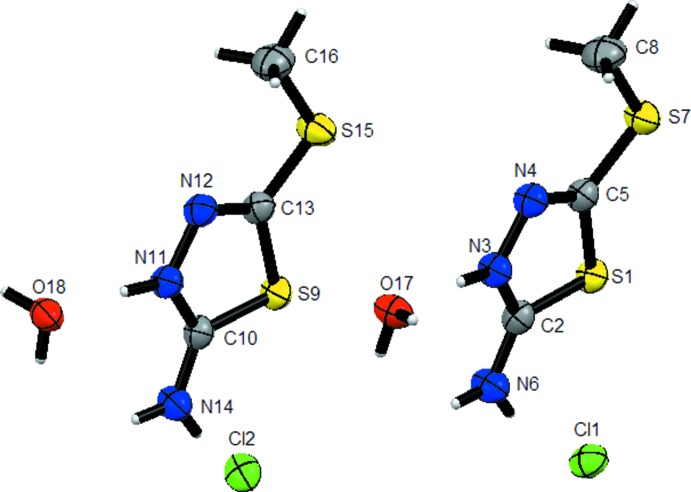
A view of molecular structure of the title salt, with atom labelling. Displacement ellipsoids are drawn at the 50% probability level.

**Fig. 2.**
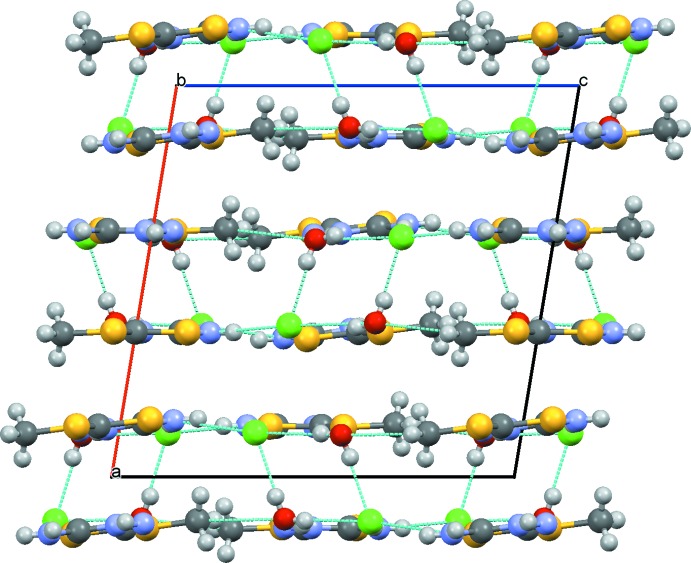
A view along the b axis of the crystal packing of the title compound. The hydrogen bonds are shown as dashed lines (see Table 1 for details).

**Fig. 3.**
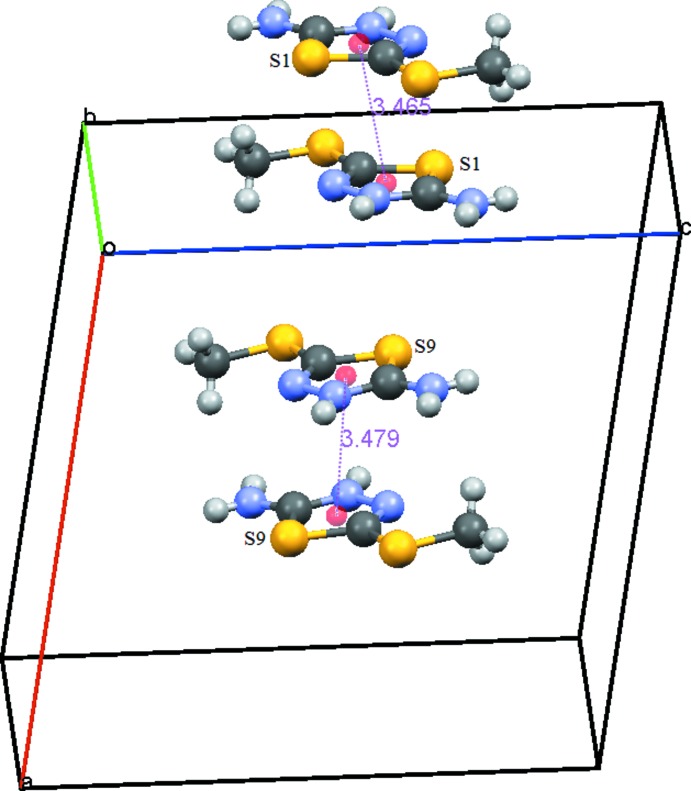
A partial view along the b axis of the crystal packing of the title compound, showing the intermolecular π–π stacking interactions between inversion-related organic cations.

. DOI: 10.1107/S1600536814015864/su2749fig1.tif
A view of mol­ecular structure of the title salt, with atom labelling. Displacement ellipsoids are drawn at the 50% probability level.

b . DOI: 10.1107/S1600536814015864/su2749fig2.tif
A view along the *b* axis of the crystal packing of the title compound. The hydrogen bonds are shown as dashed lines (see Table 1 for details).

b . DOI: 10.1107/S1600536814015864/su2749fig3.tif
A partial view along the *b* axis of the crystal packing of the title compound, showing the inter­molecular π–π stacking inter­actions between inversion-related organic cations.

CCDC reference: 1012703

Additional supporting information:  crystallographic information; 3D view; checkCIF report

## Figures and Tables

**Table 1:** Hydrogen-bond geometry (Å, °)

*D*—H⋯*A*	*D*—H	H⋯*A*	*D*⋯*A*	*D*—H⋯*A*
N3—H3⋯O17	0.861 (13)	1.818 (13)	2.6778 (7)	176.4 (12)
N6—H6*A*⋯Cl1	0.846 (12)	2.296 (12)	3.1178 (6)	164.2 (11)
N6—H6*B*⋯Cl2^i^	0.829 (13)	2.392 (13)	3.2139 (7)	171.4 (13)
N11—H11⋯O18	0.914 (14)	1.751 (14)	2.6632 (7)	176.7 (13)
N14—H14*A*⋯Cl2	0.877 (12)	2.289 (12)	3.1287 (6)	160.3 (11)
N14—H14*B*⋯Cl1^ii^	0.807 (13)	2.461 (13)	3.2648 (6)	173.6 (13)
O17—H17*A*⋯Cl2^i^	0.796 (14)	2.373 (14)	3.1593 (5)	169.8 (14)
O17—H17*B*⋯Cl2^iii^	0.826 (15)	2.340 (15)	3.1649 (6)	175.5 (14)
O18—H18*A*⋯Cl1^ii^	0.780 (13)	2.414 (13)	3.1708 (5)	163.8 (13)
O18—H18*B*⋯Cl1^iv^	0.837 (15)	2.318 (15)	3.1517 (6)	174.1 (14)

Symmetry codes: (i) 
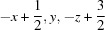
; (ii) 
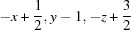
; (iii) 
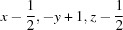
; (iv) 
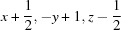
.

## supplementary crystallographic information

### S1. Chemical context

The amine under investigation, 2-amino-5-(methyl­thio)-1,3,4-thia­diazole, belongs to the larger family of heterocyclic compounds with potential medicinal importance and broad spectra of biological activities. Among these types of organic molecules are several isomers of thia­diazole (Demirbas *et al.*, 2009). 1,3,4-thia­diazo­les represent one of the most biologically active classes of compounds with a wide spectrum of activities. A large number of 1,3,4-thia­diazo­les have been patented in the agricultural field as herbicides and bactericides (Wei *et al.*, 2006). In the present investigation, we report the synthesis and crystal structure of a new organic-inorganic hybrid compound prepared from the reaction of the title amine with the hydro­chloric acid in aqueous medium.

### S2. Structural commentary

As shown in Fig. 1, the asymmetric unit of the title compound contains two 2-amino-5-(methyl­thio)-1,3,4-thia­diazol-3-ium cations, two chloride anions and two water molecules. These entities are connected by N—H···Cl, N—H···O and O—H···Cl hydrogen bonds, forming sheets parallel to (100) (Table 1 and Fig. 2). The layers are connect through O—H···Cl contacts, forming bilayers (Figs. 2 and 3).

Examination of the geometrical features of the organic cations shows that the exocyclic C—N bond lengths are similar in length to those of the thia­diazole rings, probably due to delocalization of the ring π density with the *p*-orbital electrons of the amino group. These C—N distances, *ca*. 1.33 Å, are compatible to that of [C_3_H_6_N_3_S_2_]H_2_PO_4_ which contains the same organic entity (Mrad *et al.*, 2012).

### S3. Supra­molecular features

The crystal packing is also influenced by inter­molecular π–π stacking inter­actions between inversion-related anti-parallel organic cations within the bilayers (Fig. 3); *Cg*1···*Cg*1^i^ = 3.4654 (4) Å [*Cg*1 is the centroid of the S1/N3/N4/C2/C5 ring; symmetry code: (i) -*x*, -y+*2*, -z+1] and *Cg*2···*Cg*2^ii^ = 3.4789 (4) Å [*Cg*2 is the centroid of the S9/N11/N12/C10/C13 ring; symmetry code: -*x*+1, -*y*+1, -*z*+1].

### S4. Synthesis and crystallization

An aqueous 1 *M* HCl solution and 2-amino-5-(methyl­thio)-1,3,4-thia­diazole in a 1:1 molar ratio were mixed and dissolved in sufficient ethanol. Crystals of the title salt grew as the ethanol evaporated at room temperature over the course of a few days.

### S5. Refinement

The N- and O-bound H atoms were located in difference Fourier maps and freely refined. The C-bound H atoms were included in calculated positions and treated as riding atoms: C—H = 0.98 Å with *U*_iso_(H) = 1.2*U*eq(C). Experimental details are given in Table 2.

### Crystal data

**Table:**

C_3_H_6_N_3_S_2_^+^·Cl^−^·H_2_O	*F*(000) = 832
*M**_r_* = 201.69	*D*_x_ = 1.586 Mg m^−^^3^
Monoclinic, *P*2/*n*	Mo *K*α radiation, λ = 0.7107 Å
Hall symbol: -P 2yac	Cell parameters from 30742 reflections
*a* = 13.3826 (2) Å	θ = 2.9–37.8°
*b* = 9.4258 (1) Å	µ = 0.89 mm^−^^1^
*c* = 13.5762 (2) Å	*T* = 110 K
β = 99.453 (1)°	Prism, colorless
*V* = 1689.27 (4) Å^3^	0.42 × 0.31 × 0.15 mm
*Z* = 8	

### Data collection

**Table:**

Agilent SuperNova (Dual, Cu at zero, Atlas) diffractometer	8394 independent reflections
Radiation source: SuperNova (Mo) X-ray Source	7745 reflections with *I* > 2σ(*I*)
Mirror monochromator	*R*_int_ = 0.021
Detector resolution: 10.4508 pixels mm^-1^	θ_max_ = 36.7°, θ_min_ = 2.9°
ω scans	*h* = −22→22
Absorption correction: analytical [*CrysAlis PRO* (Agilent, 2012), using a multi-faceted crystal model based on expressions derived by Clark & Reid (1995)]	*k* = −15→15
*T*_min_ = 0.769, *T*_max_ = 0.894	*l* = −22→22
65950 measured reflections	

### Refinement

**Table:**

Refinement on *F*^2^	Secondary atom site location: difference Fourier map
Least-squares matrix: full	Hydrogen site location: inferred from neighbouring sites
*R*[*F*^2^ > 2σ(*F*^2^)] = 0.020	H atoms treated by a mixture of independent and constrained refinement
*wR*(*F*^2^) = 0.054	*w* = 1/[σ^2^(*F*_o_^2^) + (0.0211*P*)^2^ + 0.370*P*] where *P* = (*F*_o_^2^ + 2*F*_c_^2^)/3
*S* = 1.12	(Δ/σ)_max_ = 0.003
8394 reflections	Δρ_max_ = 0.47 e Å^−^^3^
224 parameters	Δρ_min_ = −0.28 e Å^−^^3^
0 restraints	Extinction correction: *SHELXL97* (Sheldrick, 2008), Fc^*^=kFc[1+0.001xFc^2^λ^3^/sin(2θ)]^-1/4^
Primary atom site location: structure-invariant direct methods	Extinction coefficient: 0.00111 (13)

### Special details

**Table:**

Geometry. All e.s.d.'s (except the e.s.d. in the dihedral angle between two l.s. planes) are estimated using the full covariance matrix. The cell e.s.d.'s are taken into account individually in the estimation of e.s.d.'s in distances, angles and torsion angles; correlations between e.s.d.'s in cell parameters are only used when they are defined by crystal symmetry. An approximate (isotropic) treatment of cell e.s.d.'s is used for estimating e.s.d.'s involving l.s. planes.
Refinement. Refinement of *F*^2^ against ALL reflections. The weighted *R*-factor *wR* and goodness of fit *S* are based on *F*^2^, conventional *R*-factors *R* are based on *F*, with *F* set to zero for negative *F*^2^. The threshold expression of *F*^2^ > 2σ(*F*^2^) is used only for calculating *R*-factors(gt) *etc*. and is not relevant to the choice of reflections for refinement. *R*-factors based on *F*^2^ are statistically about twice as large as those based on *F*, and *R*-factors based on ALL data will be even larger.

### Fractional atomic coordinates and isotropic or equivalent isotropic displacement parameters (Å^2^)

**Table:**

	*x*	*y*	*z*	*U*_iso_*/*U*_eq_	
S7	0.127439 (13)	1.268596 (18)	0.444671 (12)	0.01782 (3)	
Cl2	0.386288 (13)	0.472130 (17)	0.838806 (12)	0.01897 (3)	
S1	0.129775 (12)	1.086700 (17)	0.628112 (12)	0.01498 (3)	
S9	0.353350 (12)	0.596116 (17)	0.572019 (12)	0.01549 (3)	
S15	0.370685 (15)	0.777122 (18)	0.392656 (13)	0.02012 (3)	
Cl1	0.112933 (13)	0.978631 (17)	0.878768 (12)	0.01911 (3)	
O18	0.39361 (4)	0.11409 (5)	0.40860 (4)	0.01921 (9)	
O17	0.10727 (4)	0.60603 (5)	0.44691 (4)	0.01852 (9)	
N12	0.38085 (4)	0.49199 (6)	0.40204 (4)	0.01616 (9)	
N3	0.12906 (4)	0.87190 (6)	0.51663 (4)	0.01608 (9)	
N6	0.13116 (5)	0.81093 (7)	0.68457 (4)	0.01909 (10)	
N11	0.37722 (4)	0.38126 (6)	0.46775 (4)	0.01566 (9)	
N14	0.35554 (5)	0.32153 (6)	0.63065 (4)	0.01771 (9)	
N4	0.12853 (4)	0.98357 (6)	0.45103 (4)	0.01622 (9)	
C10	0.36248 (5)	0.41383 (6)	0.55932 (5)	0.01406 (9)	
C5	0.12929 (5)	1.10218 (7)	0.49890 (5)	0.01482 (9)	
C13	0.36960 (5)	0.61102 (7)	0.44630 (5)	0.01494 (10)	
C2	0.13012 (5)	0.90405 (7)	0.61203 (5)	0.01490 (10)	
C16	0.38878 (6)	0.72871 (9)	0.26821 (5)	0.02396 (13)	
H16B	0.4528	0.6771	0.2715	0.036*	
H16A	0.3906	0.8146	0.2279	0.036*	
H16C	0.3327	0.6681	0.2376	0.036*	
C8	0.13379 (6)	1.21799 (9)	0.31782 (5)	0.02475 (13)	
H8B	0.1962	1.1643	0.3161	0.037*	
H8A	0.1335	1.3033	0.2765	0.037*	
H8C	0.0751	1.1589	0.2919	0.037*	
H18B	0.4504 (11)	0.0902 (15)	0.3960 (11)	0.045 (4)*	
H18A	0.3831 (10)	0.0703 (14)	0.4546 (10)	0.033 (3)*	
H6A	0.1303 (9)	0.8410 (13)	0.7431 (9)	0.028 (3)*	
H14B	0.3640 (10)	0.2376 (14)	0.6235 (10)	0.033 (3)*	
H14A	0.3506 (9)	0.3519 (14)	0.6907 (9)	0.031 (3)*	
H6B	0.1286 (10)	0.7247 (14)	0.6724 (10)	0.036 (3)*	
H17B	0.0504 (11)	0.5876 (15)	0.4157 (11)	0.045 (4)*	
H17A	0.1117 (10)	0.5630 (15)	0.4979 (10)	0.037 (3)*	
H3	0.1242 (9)	0.7870 (14)	0.4929 (9)	0.033 (3)*	
H11	0.3851 (10)	0.2905 (15)	0.4467 (10)	0.043 (4)*	

### Atomic displacement parameters (Å^2^)

**Table:**

	*U*^11^	*U*^22^	*U*^33^	*U*^12^	*U*^13^	*U*^23^
S7	0.02231 (7)	0.01564 (6)	0.01558 (6)	−0.00113 (5)	0.00333 (5)	−0.00038 (5)
Cl2	0.02398 (7)	0.01862 (7)	0.01352 (6)	0.00121 (5)	0.00077 (5)	−0.00314 (5)
S1	0.01744 (6)	0.01459 (6)	0.01294 (6)	−0.00041 (5)	0.00259 (5)	−0.00314 (5)
S9	0.01931 (7)	0.01389 (6)	0.01357 (6)	0.00047 (5)	0.00357 (5)	−0.00272 (5)
S15	0.02861 (8)	0.01529 (7)	0.01681 (7)	−0.00147 (6)	0.00476 (6)	0.00022 (5)
Cl1	0.02353 (7)	0.01790 (6)	0.01740 (6)	−0.00308 (5)	0.00778 (5)	−0.00374 (5)
O18	0.0268 (2)	0.01509 (19)	0.0167 (2)	0.00361 (17)	0.00633 (18)	0.00171 (16)
O17	0.0251 (2)	0.0155 (2)	0.01450 (19)	−0.00279 (17)	0.00197 (17)	0.00079 (15)
N12	0.0187 (2)	0.0157 (2)	0.0147 (2)	−0.00090 (17)	0.00432 (17)	−0.00181 (17)
N3	0.0205 (2)	0.0144 (2)	0.0132 (2)	−0.00115 (17)	0.00220 (17)	−0.00289 (16)
N6	0.0265 (3)	0.0163 (2)	0.0143 (2)	−0.0013 (2)	0.00311 (19)	−0.00122 (18)
N11	0.0194 (2)	0.0141 (2)	0.0139 (2)	−0.00025 (17)	0.00404 (17)	−0.00234 (16)
N14	0.0241 (2)	0.0152 (2)	0.0137 (2)	0.00121 (19)	0.00275 (18)	−0.00082 (17)
N4	0.0190 (2)	0.0158 (2)	0.0137 (2)	−0.00094 (17)	0.00235 (17)	−0.00223 (17)
C10	0.0144 (2)	0.0141 (2)	0.0135 (2)	0.00004 (17)	0.00170 (18)	−0.00237 (17)
C5	0.0149 (2)	0.0160 (2)	0.0135 (2)	−0.00038 (18)	0.00204 (18)	−0.00209 (18)
C13	0.0159 (2)	0.0150 (2)	0.0140 (2)	−0.00095 (18)	0.00286 (18)	−0.00155 (18)
C2	0.0153 (2)	0.0154 (2)	0.0139 (2)	−0.00111 (18)	0.00202 (18)	−0.00291 (18)
C16	0.0260 (3)	0.0305 (4)	0.0159 (3)	0.0014 (3)	0.0047 (2)	0.0019 (2)
C8	0.0297 (3)	0.0296 (4)	0.0161 (3)	−0.0003 (3)	0.0069 (2)	0.0003 (2)

### Geometric parameters (Å, º)

**Table:**

S7—C5	1.7312 (7)	N3—H3	0.861 (13)
S7—C8	1.8024 (7)	N6—C2	1.3176 (9)
S1—C2	1.7355 (6)	N6—H6A	0.846 (12)
S1—C5	1.7593 (6)	N6—H6B	0.829 (13)
S9—C10	1.7331 (6)	N11—C10	1.3264 (8)
S9—C13	1.7613 (6)	N11—H11	0.913 (14)
S15—C13	1.7278 (7)	N14—C10	1.3164 (9)
S15—C16	1.8043 (8)	N14—H14B	0.808 (13)
O18—H18B	0.836 (15)	N14—H14A	0.876 (13)
O18—H18A	0.780 (14)	N4—C5	1.2923 (8)
O17—H17B	0.827 (15)	C16—H16B	0.9800
O17—H17A	0.796 (14)	C16—H16A	0.9800
N12—C13	1.2932 (8)	C16—H16C	0.9800
N12—N11	1.3791 (8)	C8—H8B	0.9800
N3—C2	1.3280 (8)	C8—H8A	0.9800
N3—N4	1.3781 (8)	C8—H8C	0.9800
			
C5—S7—C8	99.61 (3)	N11—C10—S9	110.27 (5)
C2—S1—C5	87.51 (3)	N4—C5—S7	124.86 (5)
C10—S9—C13	87.75 (3)	N4—C5—S1	115.35 (5)
C13—S15—C16	100.21 (3)	S7—C5—S1	119.78 (4)
H18B—O18—H18A	108.1 (13)	N12—C13—S15	125.50 (5)
H17B—O17—H17A	105.6 (13)	N12—C13—S9	115.05 (5)
C13—N12—N11	109.69 (5)	S15—C13—S9	119.45 (4)
C2—N3—N4	117.01 (5)	N6—C2—N3	125.04 (6)
C2—N3—H3	124.4 (8)	N6—C2—S1	124.52 (5)
N4—N3—H3	118.4 (8)	N3—C2—S1	110.44 (5)
C2—N6—H6A	118.6 (8)	S15—C16—H16B	109.5
C2—N6—H6B	120.6 (9)	S15—C16—H16A	109.5
H6A—N6—H6B	120.6 (12)	H16B—C16—H16A	109.5
C10—N11—N12	117.23 (5)	S15—C16—H16C	109.5
C10—N11—H11	123.5 (9)	H16B—C16—H16C	109.5
N12—N11—H11	119.3 (9)	H16A—C16—H16C	109.5
C10—N14—H14B	122.1 (9)	S7—C8—H8B	109.5
C10—N14—H14A	119.6 (8)	S7—C8—H8A	109.5
H14B—N14—H14A	117.7 (12)	H8B—C8—H8A	109.5
C5—N4—N3	109.70 (5)	S7—C8—H8C	109.5
N14—C10—N11	125.16 (6)	H8B—C8—H8C	109.5
N14—C10—S9	124.57 (5)	H8A—C8—H8C	109.5
			
C13—N12—N11—C10	0.74 (8)	C2—S1—C5—S7	179.35 (4)
C2—N3—N4—C5	0.04 (8)	N11—N12—C13—S15	179.19 (5)
N12—N11—C10—N14	178.94 (6)	N11—N12—C13—S9	−0.16 (7)
N12—N11—C10—S9	−0.95 (7)	C16—S15—C13—N12	0.81 (7)
C13—S9—C10—N14	−179.23 (6)	C16—S15—C13—S9	−179.87 (4)
C13—S9—C10—N11	0.67 (5)	C10—S9—C13—N12	−0.29 (5)
N3—N4—C5—S7	−179.17 (5)	C10—S9—C13—S15	−179.68 (4)
N3—N4—C5—S1	−0.44 (7)	N4—N3—C2—N6	−179.72 (6)
C8—S7—C5—N4	−3.94 (7)	N4—N3—C2—S1	0.37 (7)
C8—S7—C5—S1	177.38 (4)	C5—S1—C2—N6	179.60 (6)
C2—S1—C5—N4	0.55 (5)	C5—S1—C2—N3	−0.49 (5)

### Hydrogen-bond geometry (Å, º)

**Table:**

*D*—H···*A*	*D*—H	H···*A*	*D*···*A*	*D*—H···*A*
N3—H3···O17	0.861 (13)	1.818 (13)	2.6778 (7)	176.4 (12)
N6—H6*A*···Cl1	0.846 (12)	2.296 (12)	3.1178 (6)	164.2 (11)
N6—H6*B*···Cl2^i^	0.829 (13)	2.392 (13)	3.2139 (7)	171.4 (13)
N11—H11···O18	0.914 (14)	1.751 (14)	2.6632 (7)	176.7 (13)
N14—H14*A*···Cl2	0.877 (12)	2.289 (12)	3.1287 (6)	160.3 (11)
N14—H14*B*···Cl1^ii^	0.807 (13)	2.461 (13)	3.2648 (6)	173.6 (13)
O17—H17*A*···Cl2^i^	0.796 (14)	2.373 (14)	3.1593 (5)	169.8 (14)
O17—H17*B*···Cl2^iii^	0.826 (15)	2.340 (15)	3.1649 (6)	175.5 (14)
O18—H18*A*···Cl1^ii^	0.780 (13)	2.414 (13)	3.1708 (5)	163.8 (13)
O18—H18*B*···Cl1^iv^	0.837 (15)	2.318 (15)	3.1517 (6)	174.1 (14)

Symmetry codes: (i) −*x*+1/2, *y*, −*z*+3/2; (ii) −*x*+1/2, *y*−1, −*z*+3/2; (iii) *x*−1/2, −*y*+1, *z*−1/2; (iv) *x*+1/2, −*y*+1, *z*−1/2.
