# Supplementary figures and images for: Crystal structure of 2-amino-5-methyl­sulfanyl-1,3,4-thia­diazol-3-ium chloride monohydrate
Source: Acta Crystallogr Sect E Struct Rep Online. 2014 Aug 1;70(Pt 9):o913–4. doi: 10.1107/S1600536814015864 (PMC4186122; doi:10.1107/S1600536814015864)

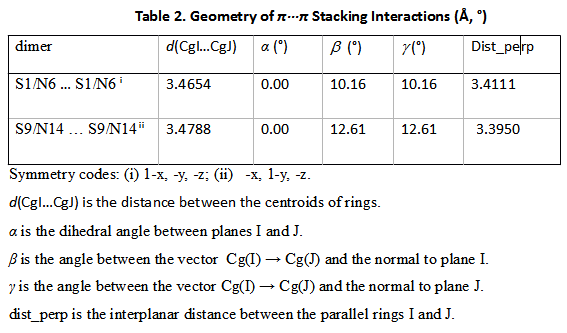

Supplement: Supplementary file 3 [file e-70-0o913-Isup3.tif]

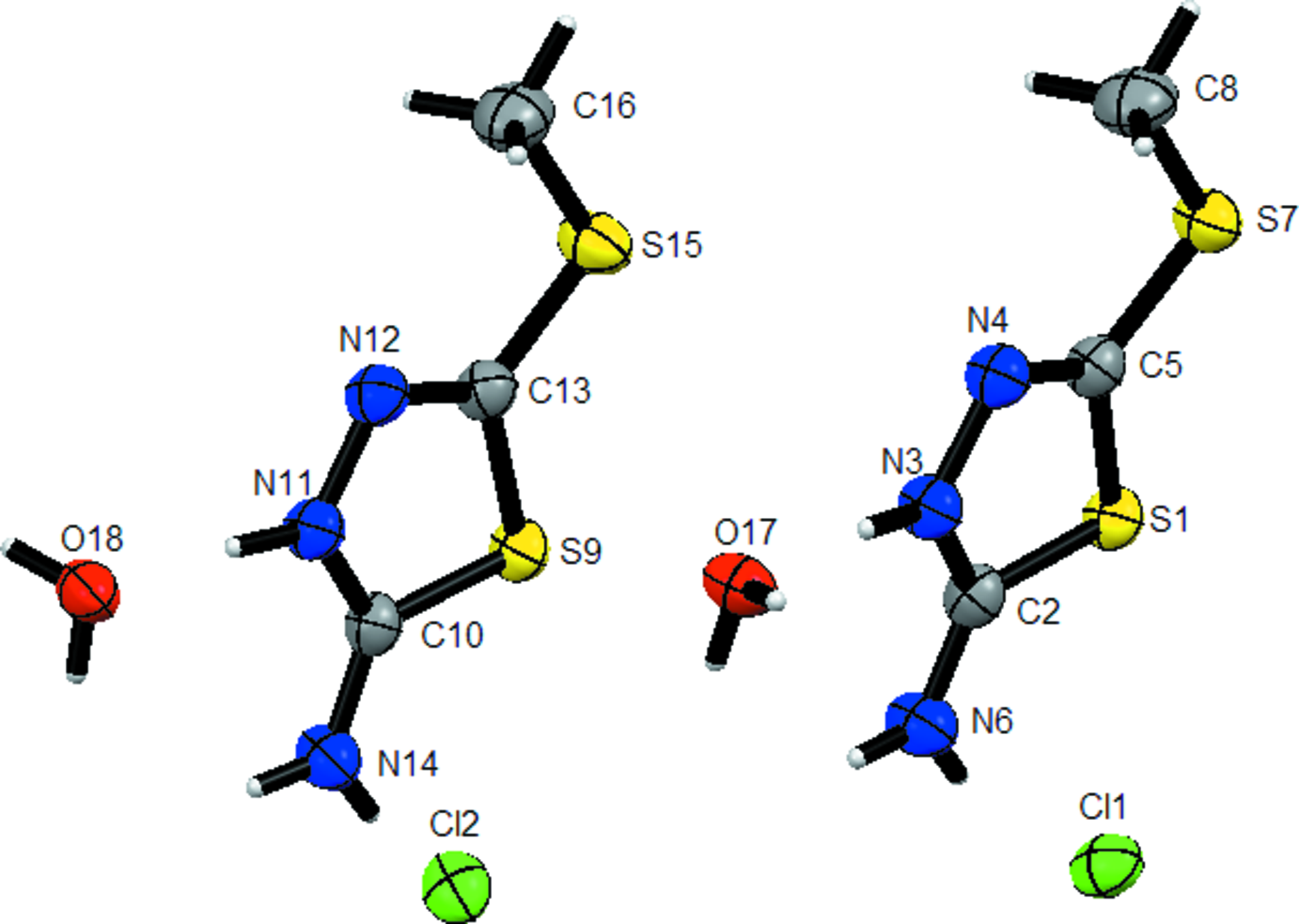

Supplement: Supplementary file 5 [file e-70-0o913-fig1.tif]

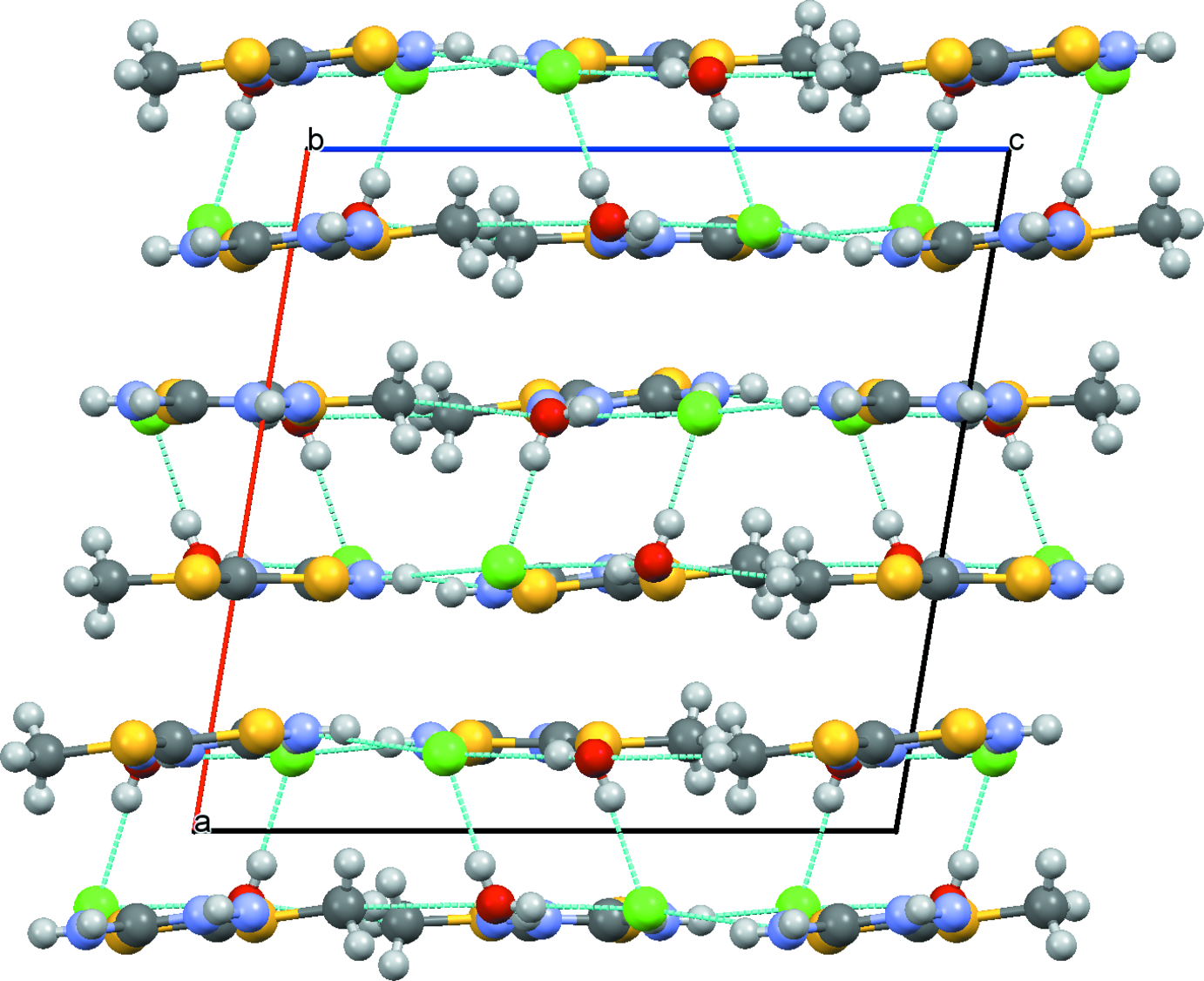

Supplement: Supplementary file 6 [file e-70-0o913-fig2.tif]

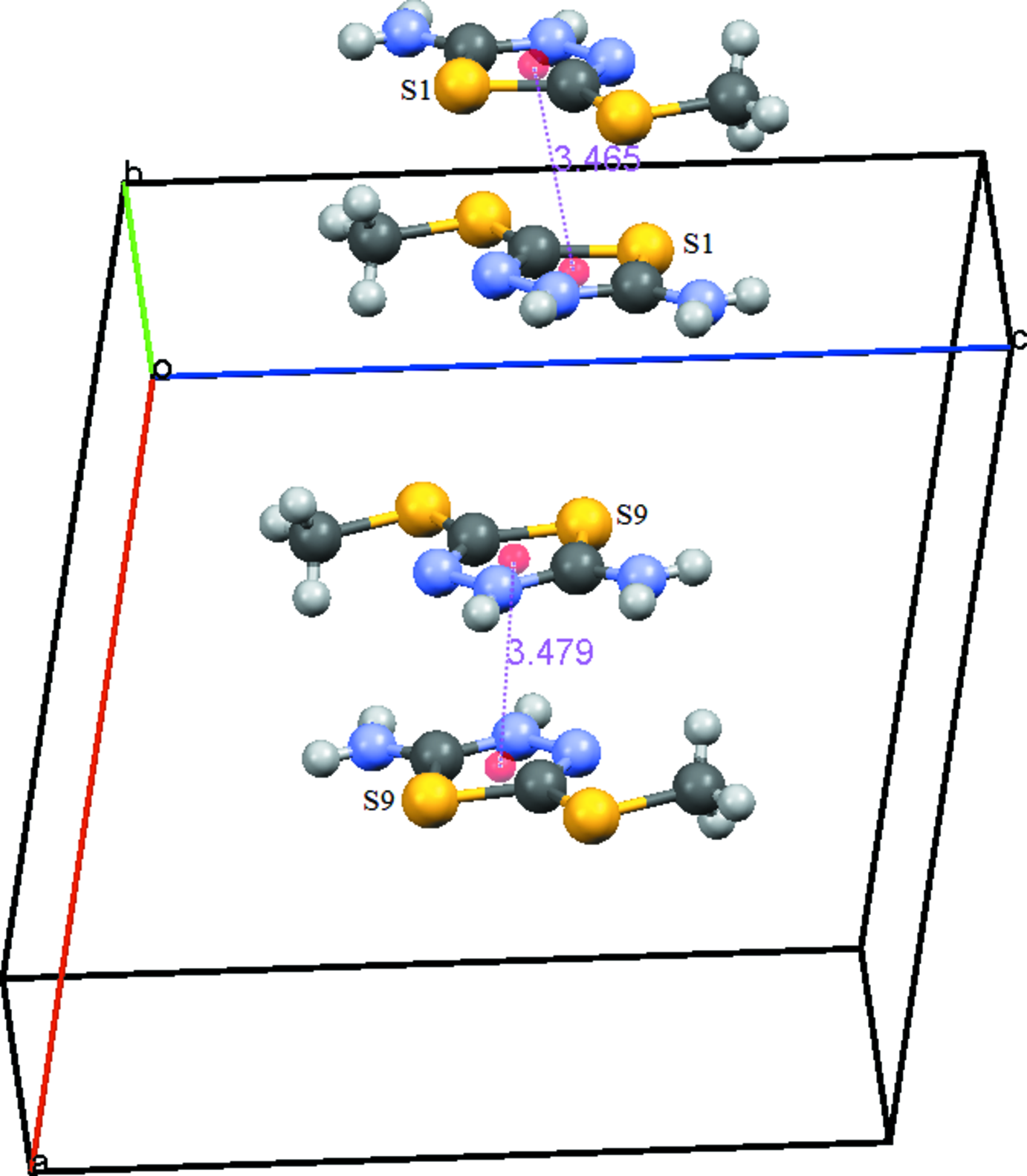

Supplement: Supplementary file 7 [file e-70-0o913-fig3.tif]
